# Hexokinase 3 promoted cytokine production of monocytes by targeting metabolic reprogramming and histone lactylation in sepsis

**DOI:** 10.1186/s13148-026-02129-6

**Published:** 2026-04-10

**Authors:** Cuiying Lian, Qizhong Xu, Hongjian Shi, Yuxuan Xie, Jianwei Fang, Boqi Rao, Tianxing Ji, Huilin Jiang, Zhuowen Wu, Xiao-Hui Chen, Shao-Peng Lin

**Affiliations:** 1 Department of Emergency, The Second Affiliated Hospital of Guangzhou Medical University, Guangzhou, 510260 People’s Republic of China; 2 State Key Laboratory of Respiratory Disease, Clinical Laboratory Medicine Department, The Second Affiliated Hospital, Guangzhou Medical University, Guangzhou, 510260 People’s Republic of China

**Keywords:** Sepsis, Monocyte, Glycolysis, Hexokinase, Histone, Lactylation

## Abstract

Sepsis is a life-threatening organ dysfunction caused by a dysregulated host immune response to infection, and there is currently a lack of early rapid identification and effective treatment methods. During the pathogenesis of sepsis, immune cells such as monocytes exhibit abnormal activation of aerobic glycolysis. However, the mechanism of glycolysis in immune cells during sepsis remains to be elucidated. Here, we investigated the role of glycolysis-related regulatory genes in the development of sepsis. Through analysis of the GEO database, we found that *HK3* is significantly elevated in the peripheral blood of sepsis patients. Receiver operating characteristic (ROC) curve analysis demonstrated that *HK3*, as a novel metabolic checkpoint, serves as an excellent diagnostic biomarker for sepsis. Immune cell infiltration analysis revealed a significant increase in monocyte infiltration in the peripheral blood of sepsis patients. Single-cell RNA sequencing analysis demonstrated a significant increase in *HK3* expression in monocytes from the sepsis group compared to the control group. Using an LPS-induced monocyte sepsis model, we found that HK3 boosts glycolytic activity and lactate accumulation. Mechanistically, this enhances inflammatory cytokine secretion through H3K18 lactylation-dependent activation of *IL-6* and *TNF-α* genes. Notably, targeted HK3 knockdown effectively suppressed this pro-inflammatory cascade, highlighting its critical role in sepsis pathogenesis. Our findings not only establish HK3 as a key metabolic regulator in sepsis but also elucidate its molecular mechanism in driving excessive monocyte-mediated inflammation. Moreover, we identify HK3 as a promising therapeutic target for mitigating hyperinflammatory responses in sepsis.

**Supplementary Information:**

The online version contains supplementary material available at 10.1186/s13148-026-02129-6.

## Introduction

Sepsis is a life-threatening organ dysfunction caused by a dysregulated host immune response to infection [1]. Global epidemiological data indicate that sepsis affects approximately 48.9 million cases annually, with a mortality rate ranging from 20 to 50%. If the condition progresses to septic shock, the mortality rate can escalate to 40%-60%. It is estimated that sepsis contributes to nearly 11 million deaths worldwide each year, accounting for 20% of global mortality—a disease burden surpassing that of malignancies and coronary heart disease. Clinical studies have demonstrated that early rapid identification and standardized intervention can significantly improve patient outcomes [2].

During the pathogenesis of sepsis, immune cells such as monocytes undergo significant metabolic reprogramming, with aberrant activation of aerobic glycolysis being particularly prominent [3]. This metabolic remodeling is closely linked to inflammatory activation and immune dysfunction in these cells. Investigating the expression changes of key glycolytic regulatory genes (e.g., *HK2*, *PKM2*, *LDHA*, *GLUT1*) not only helps elucidate the mechanistic connection between early-stage hyperglycolysis and cytokine storm in sepsis but may also provide new insights for early diagnostic biomarker screening and the development of metabolic intervention targets [4–6].

This study is the first to reveal hexokinase 3 (HK3) as a novel metabolic checkpoint in sepsis, playing a critical role in its pathogenesis. We found that HK3 in monocytes significantly promotes the expression of pro-inflammatory genes and the secretion of inflammatory cytokines by regulating energy metabolic reprogramming and histone H3 lysine 18 lactylation (H3K18la). Conversely, specific knockdown of HK3 effectively suppresses this pro-inflammatory process. Our findings not only provide a new perspective on the molecular mechanisms of sepsis but also establish a theoretical foundation for developing metabolism-targeted precision therapies.

## Experimental methods

### Data acquisition and identification of differentially expressed genes (DEGs)

We searched the Gene Expression Omnibus (GEO) database (https://www.ncbi.nlm.nih.gov/geo/) from the National Center for Biotechnology Information (NCBI) using "sepsis" as the keyword to identify sepsis-related datasets. After screening, we selected dataset GSE100159 for analysis. This dataset was submitted by Altman MC in 2020 and was generated using the Illumina HumanWG-6 v3.0 expression beadchip platform [7]. It consists of whole blood samples from 35 sepsis patients and 12 healthy controls. To ensure homogeneity within the sepsis group, two sepsis-recovery patient samples were excluded from our study. Heatmap, principal component analysis, and volcano plot was plotted by https://www.bioinformatics.com.cn, an online platform for data analysis and visualization.

Sixty-two glycolysis-related genes (GRGs) were acquired from the Molecular Signatures Database of Gene Set Enrichment Analysis (GSEA) (http://www.gsea-msigdb.org/gsea/index.jsp). By intersecting the GSE100159 dataset with GRGs, we identified differentially expressed GRGs between sepsis patients and healthy controls. A threshold of *P* < 0.05 and |Log2 Fold change|> 1 was set for screening differentially expressed genes.

### Screening of potential diagnostic markers of sepsis

The MedCalc software package (version 22.023; MedCalc Software Ltd, Ostend, Belgium) was applied to search for biomarkers with the best diagnostic value among the differentially expressed GRGs. The efficacy of genes in distinguishing sepsis from healthy control was measured via the area under the curve (AUC).

### Immune infiltration analysis

Cell-type Identification by Estimating Relative Subsets of RNA Transcripts (CIBERSORT) is a deconvolution algorithm based on the principle of linear support vector regression to study the expression matrix of immune cell subtypes [8]. The immune cells composition of healthy subjects and patients with sepsis was analyzed and plotted by CIBERSORT in https://www.bioinformatics.com.cn.

### Single-cell RNA sequencing analysis

The single-cell transcriptomics dataset GSE175453 was downloaded from the GEO database [9]. This dataset comprises 5 control group samples and 4 sepsis patient samples. The Seurat package was used for data preprocessing, and the Harmony package (Version 1.2.4) was applied to correct batch effects under default parameters. Based on the filtered principal components, cell clustering was performed using the FindNeighbors and FindClusters functions. Subsequently, the Uniform Manifold Approximation and Projection (UMAP) method was utilized to visualize the clustering results. The SingleR package was employed to annotate each cell cluster, and the annotation results were displayed via UMAP. Finally, the expression levels of the *HK3* gene across different cell types were compared between the control group and sepsis patients.

### Isolation and culture of primary human monocytes

Healthy donors were included and approximately 2 mL of peripheral blood samples were obtained in EDTA anticoagulant tubes. Human peripheral blood mononuclear cells (PBMCs) were isolated from fresh peripheral blood of healthy donors using Ficoll gradient centrifugation [10]. Briefly, diluted blood was layered over Ficoll and centrifuged at 400 g for 30 min. The PBMC layer was collected, washed twice with PBS, and resuspended in complete RPMI-1640 medium. Monocytes were then purified from the PBMCs by differential adhesion. The cell suspension was seeded at 5 × 10^6^ cells/ml in 6-well plates and incubated for 2 h at 37 °C in 5% CO₂, after which non-adherent cells were removed by extensive washing with warm PBS. The resulting adherent cell population was used for subsequent experiments. An inflammation model was established by stimulating the cells with 125 ng/mL lipopolysaccharide (LPS; Sigma, USA) for 24 h. Our studies were approved by the Ethics Committee of the Second Affiliated Hospital of Guangzhou Medical University (2024-YJS-ks-14).

### Cell culture of RAW264.7 cells

The RAW264.7 cell line were purchased from Procell Life Science & Technology (Wuhan, China). RAW264.7 cells were seeded in culture dishes containing complete medium supplemented with 10% fetal bovine serum (FBS) and incubated at 37 °C in a humidified atmosphere with 5% CO₂. A sepsis cell model was established by stimulating the cells with LPS.

### Cell transfection

The siRNA targeting the *HK3* gene and the negative control (scrambled siRNA) were synthesized by Sangon Biotech Co., Ltd. (Shanghai, China). RAW264.7 cells in the logarithmic growth phase were seeded into 6 well plates. Subsequently, the cells were transfected with either a scrambled siRNA (si-NC) or HK3 siRNA (si-HK3) for a duration of 24 h using Lipofectamine 3000 reagent (Invitrogen, USA) and Opti-MEM reduced serum medium (Gibco, USA). Sequences of siRNA can be found in Table 1.

**Table 1:** Sequences of siRNA

Names		Sequence (5’-3’)
*si-HK3*	Sense	CUCAAAGGUUUGAGAAGAUTT
	Antisense	AUCUUCUCAAACCUUUGAGTT
*si-NC*	Sense	UUCUCCGAACGUGUCACGUTT
	Antisense	UUCUCCGAACGUGUCACGUTT

### Enzyme-linked immunosorbent assay (ELISA) assay

The levels of Il-6 and TNF-α in the cell culture supernatant were analyzed using ELISA kits (EH0201 and EH0302, FineTest, Hubei, China for primary human monocytes experiments; EM0004 and EM0010, Youke Life Sciences Techonology CO., LTD. Hangzhou, China for RAW264.7 cell experiments), in accordance with the manufacturer’s instructions. The absorbance at 450 nm was measured by a microplate reader (Tecan, USA). The standard curve was generated based on the measured absorbance values, and the sample concentrations were calculated accordingly.

### Quantitative real-time polymerase chain reaction (qRT-PCR)

Total RNA was extracted from cells using the EZ-press RNA Purification Kit (EZBioscience, USA). Subsequently, total RNA was reverse transcribed into cDNA using the SuperScript III Reverse Transcription Kit (Invitrogen, USA) according to the provided instructions [11]. The PCR amplification was thereafter performed using SYBR Green qPCR SuperMix in a ViiA ™7 Real-Time PCR System (Applied Biosystems, USA). The results were calculated using the 2^−ΔΔCt^ method. The primer sequences for all genes can be found in Table 2.

**Table 2:** qRT-PCR primer sequences

Primers		Sequence (5’-3’)
*HK3*	Forward	ATTTCGGTTAAGTGGCTACAGAGG
	Reverse	TGCTGCAAGCATTCCAGTTCTA
*IL-6*	Forward	TAGTCCTTCCTACCCCAATTTCC
	Reverse	TTGGTCCTTAGCCACTCCTTC
*TNF-α*	Forward	GGTCCCCAAAGGGATGAGAAGT
	Reverse	TTGCTACGACGTGGGCTACA
*β-actin*	Forward	CATGTACGTTGCTATCCAGGC
	Reverse	CTCCTTAATGTCACGCACGAT

### Immunofluorescence staining

For immunofluorescence staining, cells were fixed with 4% paraformaldehyde and permeabilized with 0.3% Triton X-100. After blocking with 5% BSA, the cells were incubated with an anti-HK3 primary antibody (Proteintech China, Cat No. 67803–1-Ig, China; 1:200), followed by an Alexa Fluor 594-conjugated secondary antibody. Nuclei were visualized with DAPI staining. Fluorescent images were captured using a ZEISS fluorescence microscope.

### Western blot analysis

Cells were lysed in RIPA buffer (Invitrogen, USA), which was supplemented with phenylmethylsulphonyl fluoride (PMSF; Thermo, USA) and phosphatase inhibitor cocktail (Beyotime, China). The protein expression levels were then analyzed using a Western blot assay as previously described [12, 13]. Briefly, proteins were separated by 12% sodium dodecyl sulfate–polyacrylamide gel electrophoresis (SDS-PAGE) and transferred to polyvinylidene fluoride (PVDF) membranes. After blocking with 5% nonfat milk, the membranes were incubated overnight at 4 °C with primary antibodies against HK3 (Proteintech China, Cat No. 67803–1-Ig, China; 1:1000), α-Tubulin (Cell Signaling, Cat No.2144S, USA; 1:1000), H3K18la (PTM BIO, Cat No.PTM-1406RM, China; 1:1000) and Histone H3 (Proteintech China, Cat No. 81984-2-RR, China; 1:25000). The membranes were then washed three times with TBST containing Tween-20 and incubated with horseradish peroxidase (HRP)-conjugated secondary antibody at room temperature for 2 h. Finally, protein expression levels were analyzed using the Image J software.

### Cell metabolism analysis

Proton Efflux Rate (PER) were measured using a Seahorse XFe96 Extracellular Flux analyzer (Agilent, USA). Cells were seeded into an XF96 cell culture plate at a density of 50,000 cells per well and incubated overnight with LPS. Then, the medium was replaced with Seahorse assay medium and the plates were incubated for 1 h in a non-CO_2_ incubator at 37 °C. Basal measurements were taken, followed by consecutive measurements executed by the sequential addition of rotenone & antimycin A (0.5 μmol/L), and the glycolysis inhibitor 2-Deoxy-D-glucose (2-DG; 50 mmol/L) to determine basal and compensatory PER. The basal PER was calculated as the average PER value during the basal measurement cycle, prior to the addition of any compounds. Compensatory Glycolysis was calculated after adding rotenone & antimycin A. Finally, PER values were normalized to the cell protein.

### Lactate content detection

Lactate levels in cell culture supernatant were measured by a colorimetric method. Briefly, cell culture supernatant was collected. Lactate levels were detected according to the instructions of the L-lactate detection kit manufacturer (A019-2-1, Nanjing Jiancheng Bioengineering Institute, China).

### Chromatin immunoprecipitation (ChIP) assay

Formaldehyde was added to the cells to cross-link nuclear proteins with genomic DNA, which was terminated by the glycine (0.125 M). Following two washes with ice-cold PBS, cells were collected, resuspended and centrifugated in ChIP lysis buffer. The supernatants were discarded and the nuclei were resuspended in nucleus lysis buffer. Lysates were sonicated at 4 °C with the pulse 5 s on and 5 s off for 3 min. The supernatants were collected after centrifugation, diluted by ChIP dilution buffer. Then the samples were divided into three parts. One part is for Input, and the other two parts are for antibodies against rabbit IgG (PTM BIO, Cat No.PTM-5073, China; 1:50) and H3K18la (PTM BIO, Cat No.PTM-1406RM, China; 1:50). Protein A/G Magnetic Beads were added to each reaction and incubated overnight at 4 °C on a rotating wheel, and then were washed by wash buffer and collected by elution buffer. The samples were de-crosslinked by adding RNase A and protease K and incubated at 62 °C for 4 h and at 95 °C for 10 min. DNA was purified using the PCR purification kit (Beyotime Biotechnology, D0033, China). The resulting DNA samples were analyzed by qPCR or semi-quantitative PCR followed by electrophoresis on an agarose gel. Primers for *IL-6* and *TNF-α* used in the analysis can be found in Table 3.

**Table 3:** ChIP- qPCR and semi-quantitative PCR primer sequence

Primers		Sequence (5’-3’)
*IL-6*	Forward	AGGTTTCCAACAGCCCCAC
	Reverse	GTTCTTGGTGGGCTCCAGAG
*TNF-α*	Forward	CGGGGAGTCATACGGATTGG
	Reverse	TGAGTTTTCCACGGAGCCTC

### Statistical analysis

Wilcoxon Rank-Sum Test was used to compare the differences in transcriptomics and immune infiltration. Statistical analysis of cell-based functional experiments was performed using GraphPad Prism 9.5 (GraphPad software, USA). Data are expressed as means ± standard deviation. Student’s *t*-test was used to compare two independent groups. One-way analysis of variance (ANOVA) with Tukey’s post hoc test was used for comparisons among multiple groups. A *P*-value < 0.05 was considered statistically significant.

## Results

### HK3 is a potential diagnostic biomarker and therapeutic target for sepsis.

**Fig. 1:**
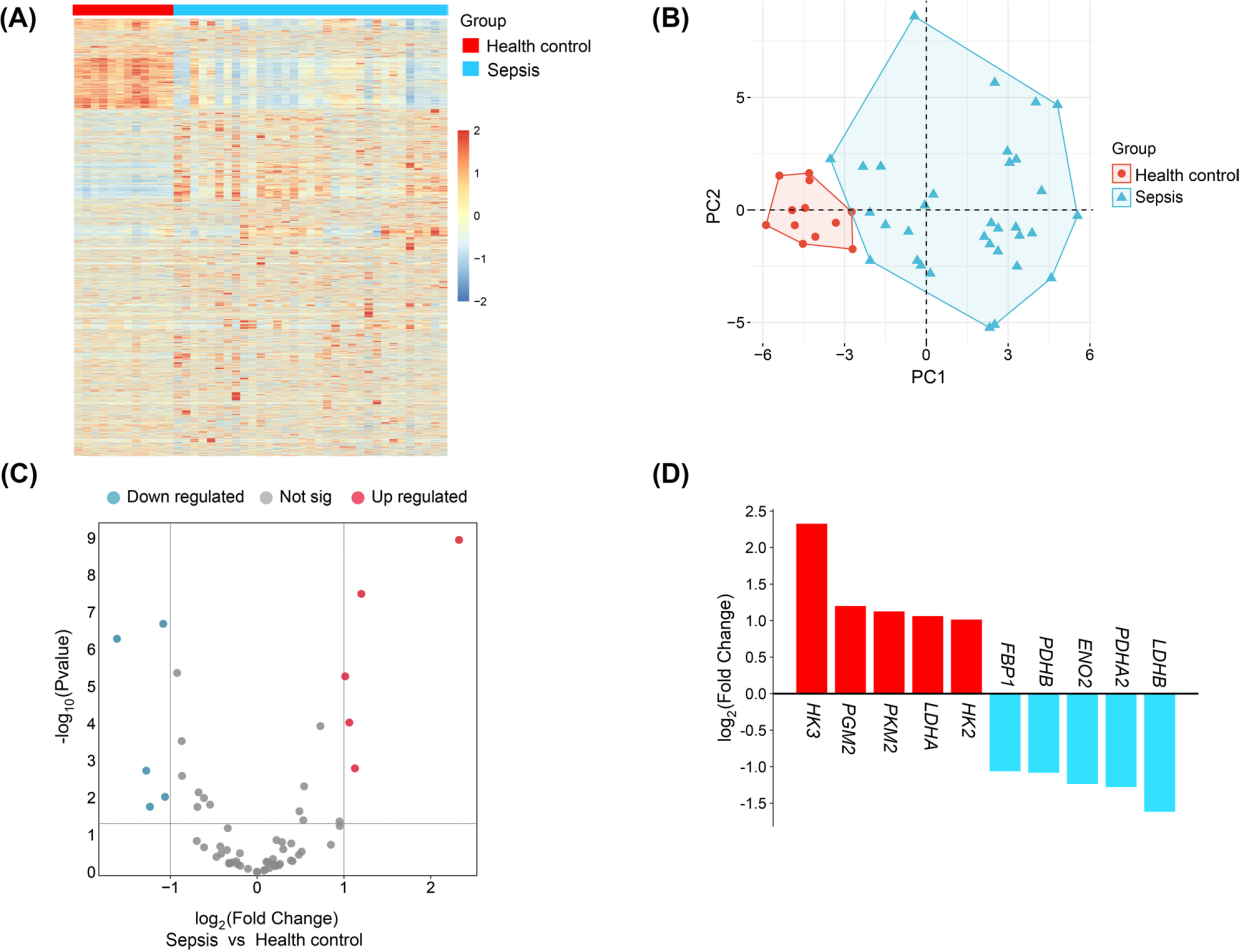
Screening sepsis related genes involved in the glycolysis pathway. **A** Expression heatmap showing differentially expressed genes between sepsis and health control. **B** Principal Component (PC) analysis of genes in sepsis patients and healthy control. **C** Volcanic diagram displays differentially expressed glycolysis-related genes. **D** Differential expression of glycolysis-related genes in peripheral blood

Analysis of the GSE100159 dataset through heatmap clustering and principal component (PC) analysis revealed significant differences in gene expression between sepsis patients and healthy controls (Fig. 1A and B). Volcano plot analysis of glycolysis-related genes showed that five genes (*HK3*, *PGM2*, *PKM2*, *LDHA*, and *HK2*) were significantly uegulated in the sepsis group, while another five genes (*FBP1*, *PDHB*, *ENO2*, *PDHA2*, and *LDHB*) were significantly downregulated. Among these, *HK3* exhibited the highest fold change, suggesting that glycolysis-related genes play a role in sepsis pathogenesis (Fig. 1C and D).

**Fig. 2:**
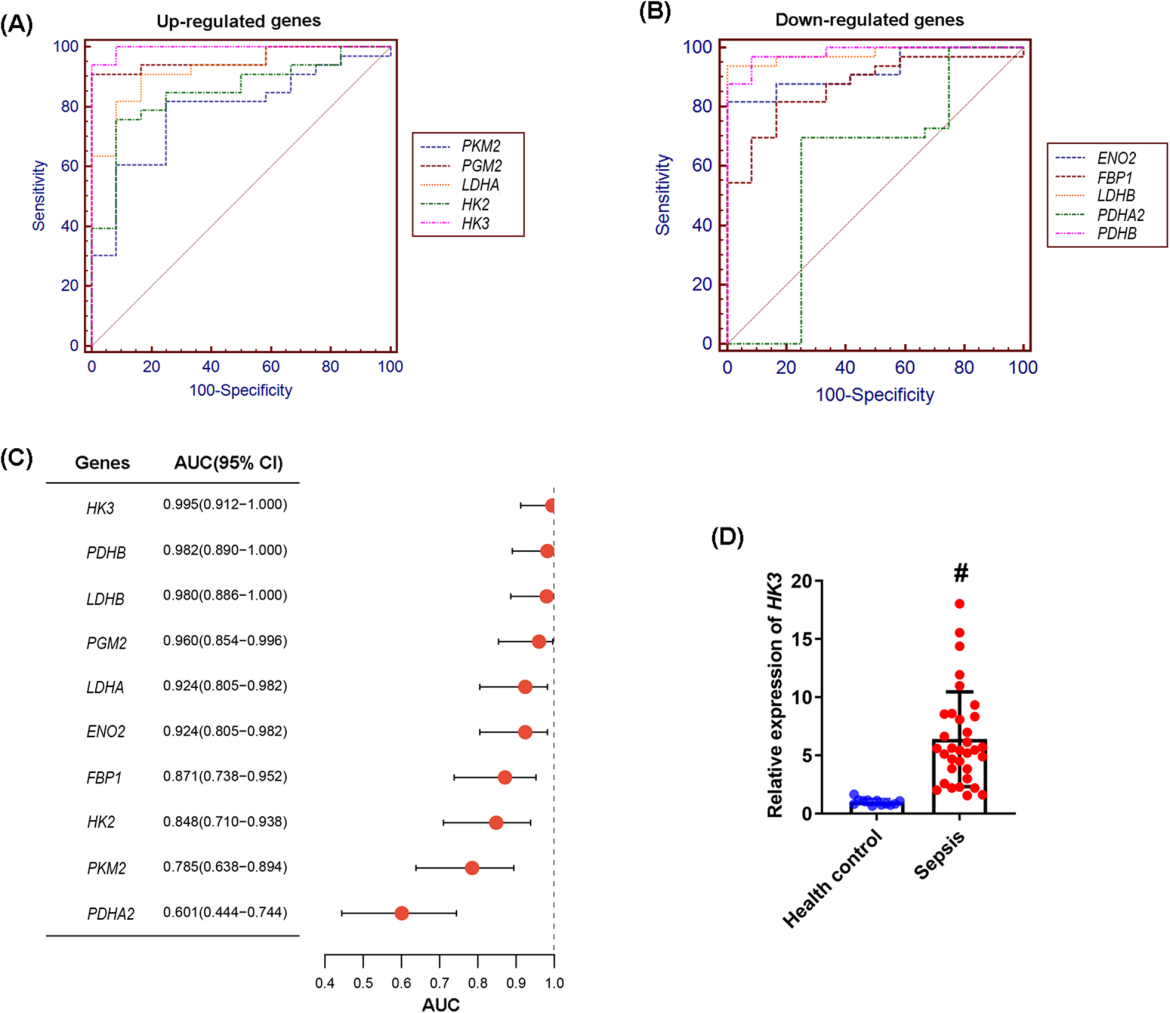
*HK3* was a potential diagnostic biomarker for sepsis. **A** ROC curve analysis of the diagnostic value of the up-regulated glycolysis-related genes. **B** ROC curve analysis of the diagnostic value of the down-regulated glycolysis-related genes. **C** Comparison of AUC among genes. **D** The expression level of *HK3* between sepsis patients and healthy control group. ^#^*P* < 0.05 compared to the healthy control group. Data were compared by Student’s *t*-test

We further evaluated the diagnostic potential of these 10 differentially expressed glycolysis-related genes in sepsis. The results demonstrated that all exhibited high diagnostic value (Fig. 2A and B). Notably, *HK3* displayed the largest area under the curve (AUC), indicating that *HK3* is a promising biomarker for sepsis diagnosis (Fig. 2C and D).

### HK3 knockdown markedly alleviated cytokine production on RAW264.7 cells.

**Fig. 3:**
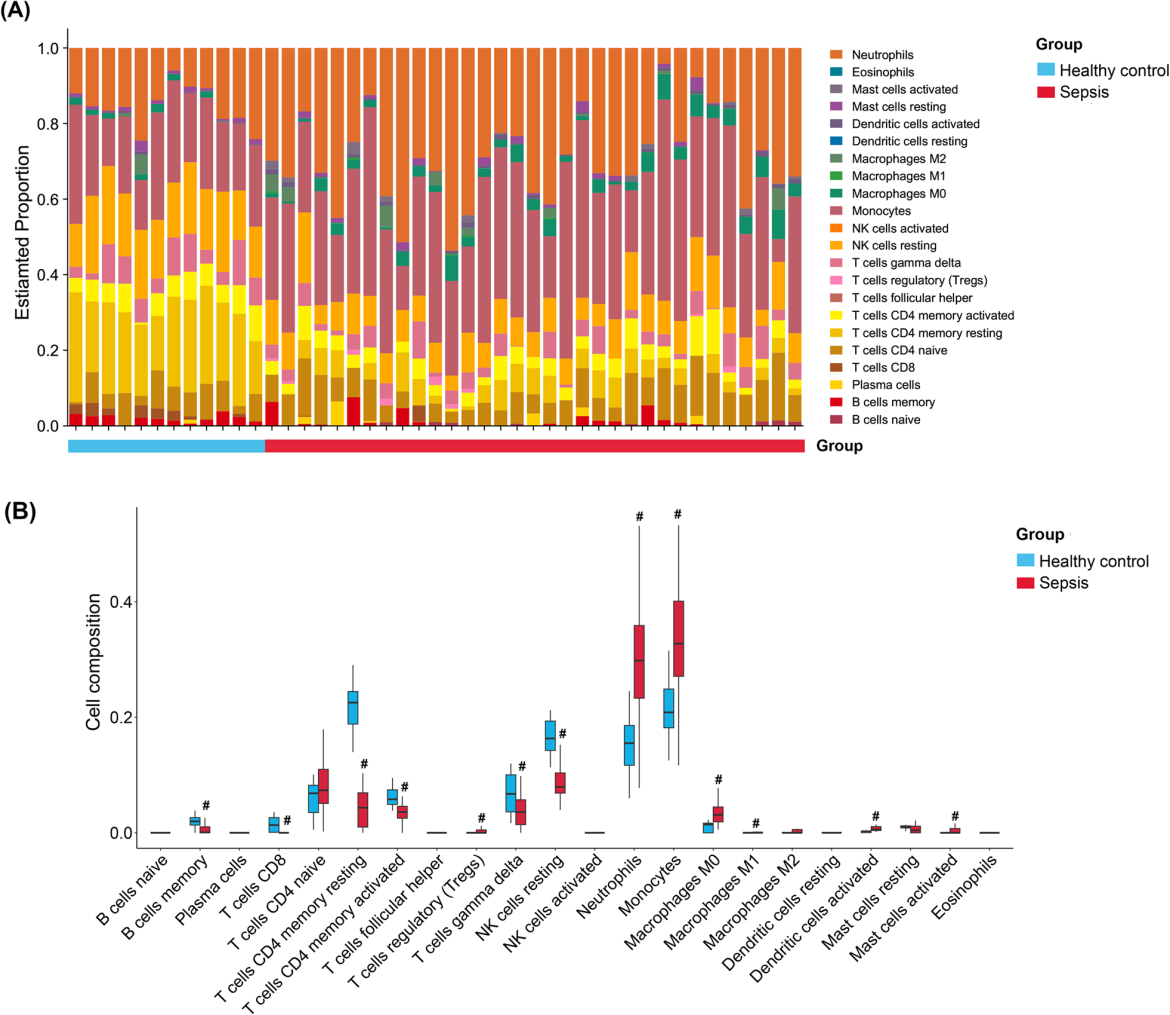
Immune infiltration Analysis. **A** The distribution of the proportions of 22 types of immune cells in all samples. **B** The immune cell infiltration analysis between the sepsis patients and healthy cohort. ^#^*P* < 0.05 compared to the healthy control group. Data were compared by Wilcoxon Rank-Sum Test

We employed the CIBERSORT algorithm to analyze immune cell infiltration in peripheral whole blood from sepsis patients. The results demonstrated a significantly higher abundance of immune cell infiltration in the sepsis group compared to controls, with a marked increase in monocyte infiltration (Fig. 3A and B).

**Fig. 4:**
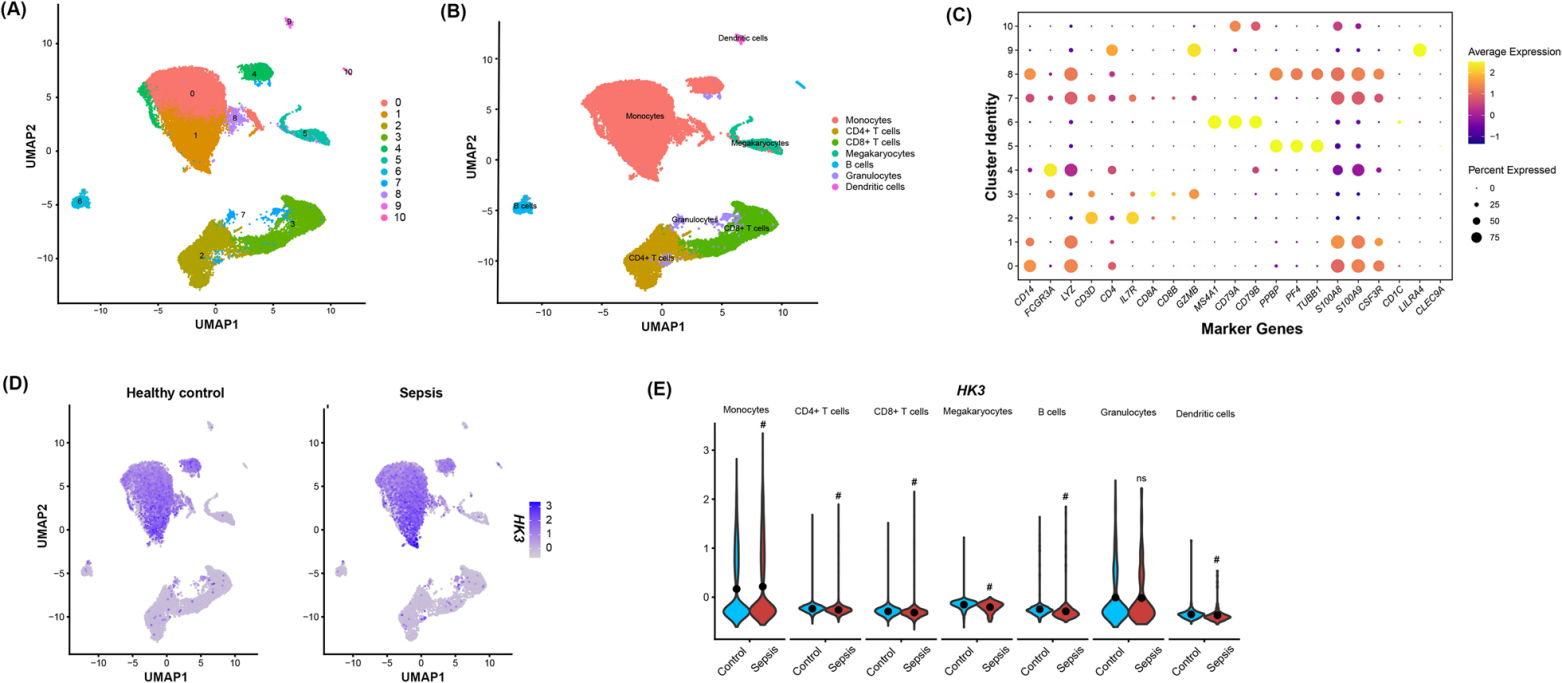
Single-cell gene expression analysis between healthy control and sepsis groups. **A** UMAP plot showed cell clusters after dimensionality reduction and clustering. **B** UMAP plot showing cell type annotations. **C** Expression levels of marker genes for each cell cluster. **D** Cellular localization of *HK3* in the healthy control and sepsis groups. **E** Comparison of *HK3* expression levels in peripheral blood mononuclear cells between the healthy control and sepsis groups. ^#^*P* < 0.05 compared to the healthy control group. Data were compared by Wilcoxon Rank-Sum Test

To further investigate the expression characteristics of *HK3* at the single-cell level, the single-cell RNA sequencing dataset GSE175453 was analyzed. First, the cell population was divided into 11 clusters by clustering analysis (Fig. 4A). Subsequently, based on the expression patterns of marker genes, these clusters were further annotated into seven distinct cell types, including monocytes, CD4^+^ T cells, CD8^+^ T cells, megakaryocytes, B cells, granulocytes, and dendritic cells (Fig. 4B). Bubble plots displayed the expression of key marker genes for each cell cluster (Fig. 4C). Comparison of *HK3* expression across different immune cell populations revealed that it was predominantly expressed in monocytes in both the control and sepsis groups (Fig. 4D). Further analysis showed that the expression level of *HK3* in monocytes was significantly increased in the sepsis group compared to the control group (Fig. 4E).

**Fig. 5:**
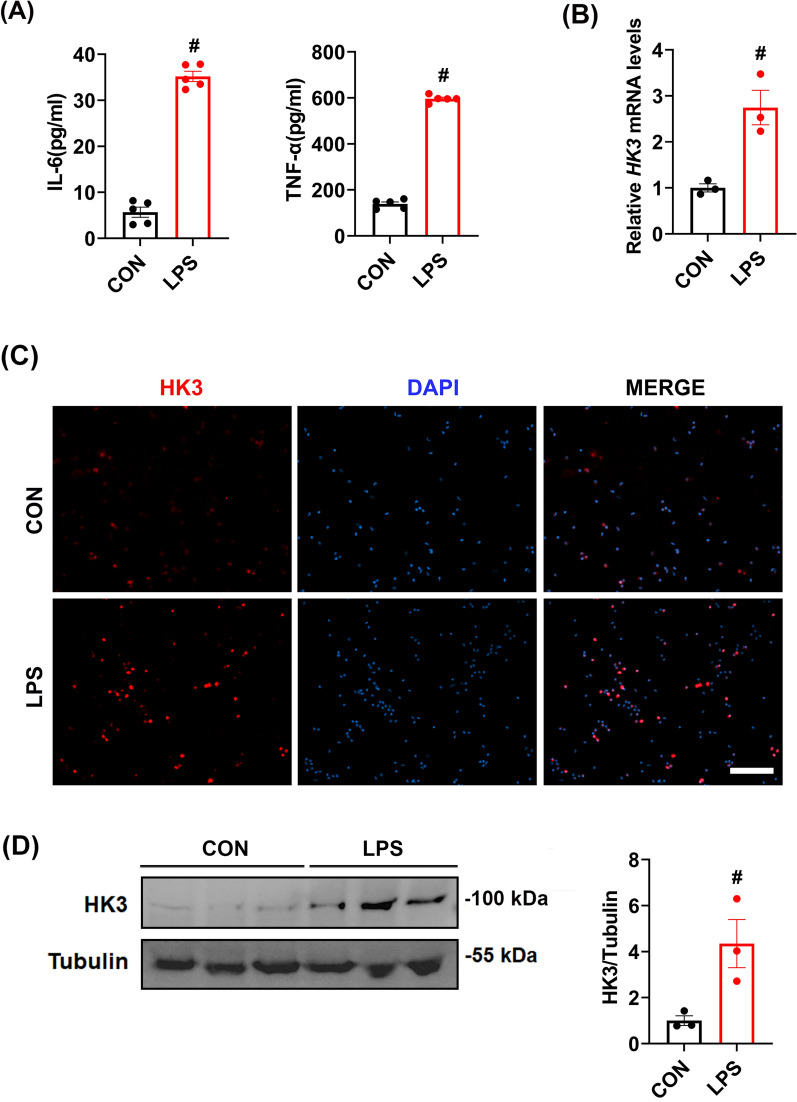
HK3 was involved in the cytokine production of human primary monocytes. **A** ELISA was used to detect the expression levels of IL-6 and TNF-α in human primary monocytes. **B** The expression level of *HK3* mRNA was measured. **C** Immunofluorescence staining was used to detect the expression of HK3. **D** The expression of HK3 protein was detected by Western blot. Scale bar = 100 μm. #*P* < 0.05 compared to the control group. Data were compared by Student’s *t*-test

LPS stimulation of primary monocytes induced the release of inflammatory cytokines (Fig. 5A). Subsequent qRT-PCR analyses revealed that LPS stimulation significantly up-regulated the mRNA of *HK3* (Fig. 5B). What is more, immunofluorescence staining and Western blot confirmed that LPS markedly enhanced HK3 expression (Fig. 5C and D).

**Fig. 6:**
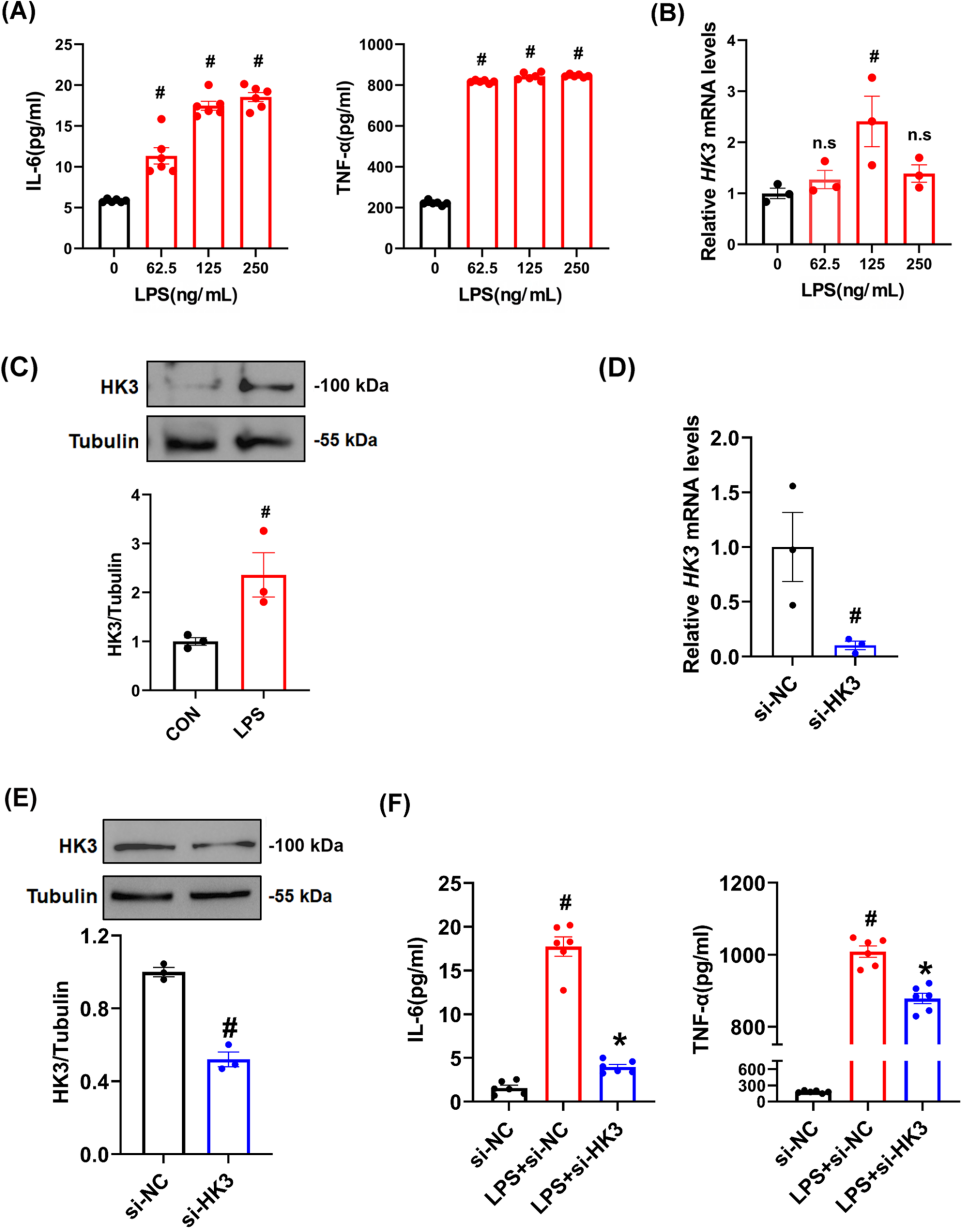
HK3 knockdown markedly alleviated cytokine production on RAW264.7 cells. **A** ELISA was used to detect the expression levels of IL-6 and TNF-α in RAW264.7 cells. **B** The expression level of *HK3* mRNA was measured. **C** The expression of HK3 protein was detected by Western blot. **D** The efficiency of si-HK3 knockdown. **E** The expression of HK3 protein was detected by Western blot. **F** ELISA was used to detect the expression levels of IL-6 and TNF-α. "ns" means *P* ≥ 0.05 compared to the control group. #*P* < 0.05 compared to the control group. *P < 0.05 compared to the LPS + si-NC group. **A**, **B**, **F** Data were compared by One-way ANOVA with Tukey’s post hoc test. **C**, **D**, **E** Data were compared by Student’s *t*-test

To validate the functional role of HK3 in monocytes, we established an in vitro sepsis model using RAW 264.7 cells (Fig. 6A–C). Subsequently, we constructed siRNA-mediated HK3 knockdown (si-HK3) and confirmed its knockdown efficiency via qRT-PCR and Western blot on RAW264.7 cell (Fig. 6D and E). ELISA assays demonstrated that si-HK3 significantly suppressed the release of inflammatory cytokines from RAW264.7 cells, indicating its potential anti-inflammatory effects (Fig. 6F).

### HK3 promoted cytokine production of RAW264.7 cells by targeting metabolic reprogramming and histone lactylation.

**Fig. 7:**
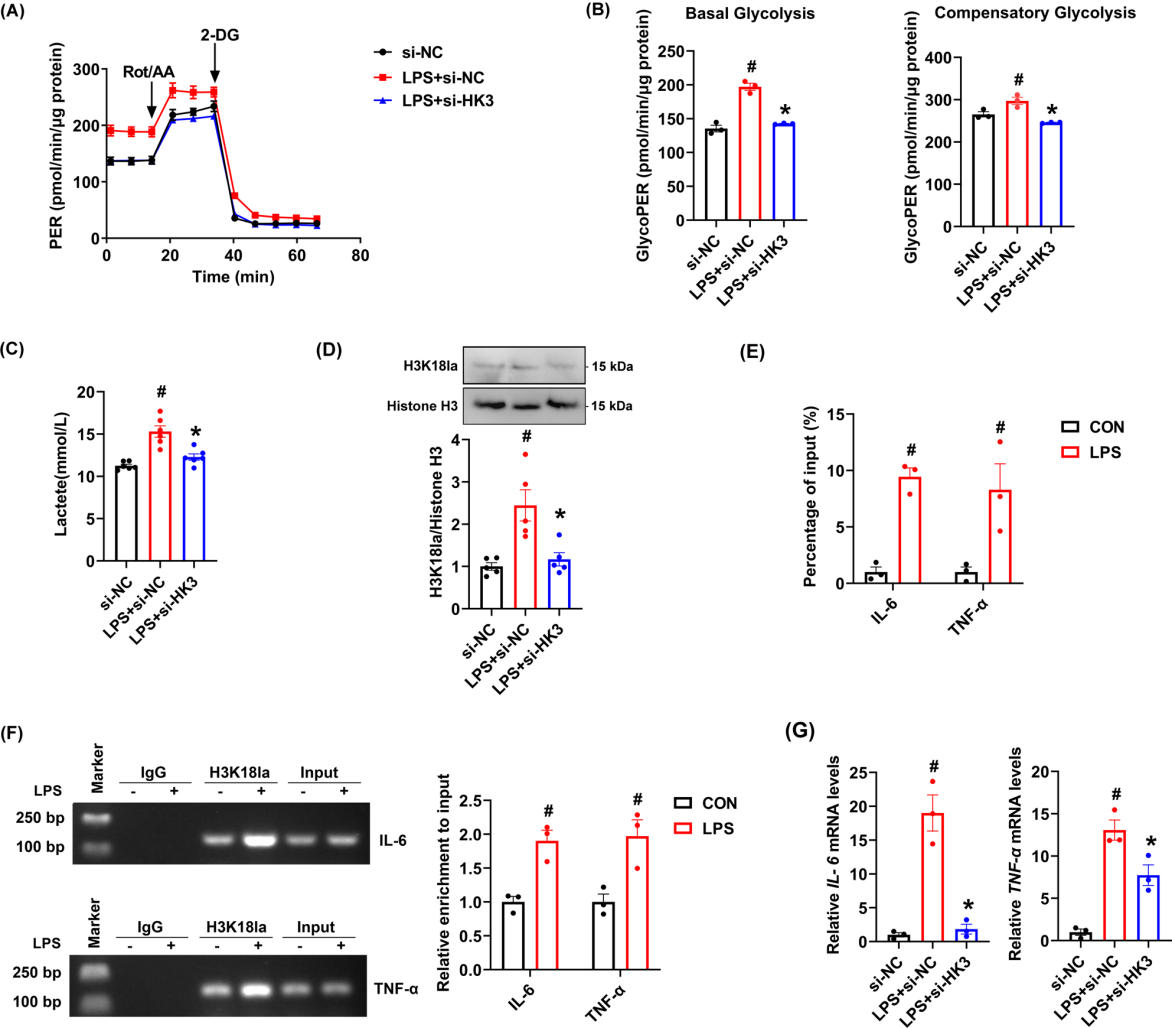
HK3 promoted cytokine production of RAW264.7 cells by targeting metabolic reprogramming and histone lactylation. **A** Proton Efflux Rate (PER) was measured by the Seahorse test in RAW264.7 cells. **B** Quantitative analysis of basal glycolysis and compensatory glycolysis. **C** Detection of lactate level. **D** The expression of H3K18la protein was detected by Western blot. **E** DNA fragments were immunoprecipitated with the H3K18la antibody and analyzed by qPCR with primers for *IL-6* and *TNF-α* gene. **F** Semi-quantitative PCR analysis of DNA prepared by ChIP. **G** qRT-PCR was used to detect the expression levels of *IL-6* and *TNF-α*. ^#^*P* < 0.01 compared to control group; *P < 0.05 compared to the LPS + si-NC group. **B**, **C**, **D**, **G** Data were compared by One-way ANOVA with Tukey’s post hoc test. **E**, **F** Data were compared by Student’s *t*-test

To elucidate the mechanism by which HK3 regulates monocyte activation, we conducted a series of experiments. First, using Seahorse extracellular flux analysis and lactate production assays, we found that LPS stimulation significantly enhanced glycolysis and lactate generation in monocytes, an effect that was dependent on HK3 expression. HK3 knockdown markedly attenuated both glycolytic activity and lactate accumulation, demonstrating HK3's crucial role in regulating metabolic reprogramming and lactate production in monocytes (Fig. 7A–C).

Further investigation revealed that LPS stimulation elevated H3K18la levels in RAW264.7 cells, while HK3 knockdown significantly suppressed this modification (Fig. 7D). ChIP-qPCR and semi-quantitative PCR analysis further revealed enrichment of H3K18la at the *IL-6* and *TNF-α* promoter in monocytes stimulated by LPS compared with control group (Fig. 7E and F). These results suggest that *IL-6* and *TNF-α* were target genes of H3K18la. Accordingly, we examined *IL-6* and *TNF-α* mRNA expression and found that LPS stimulation upregulated both mRNA, whereas HK3 knockdown substantially inhibited their expression (Fig. 7G).

**Fig. 8:**
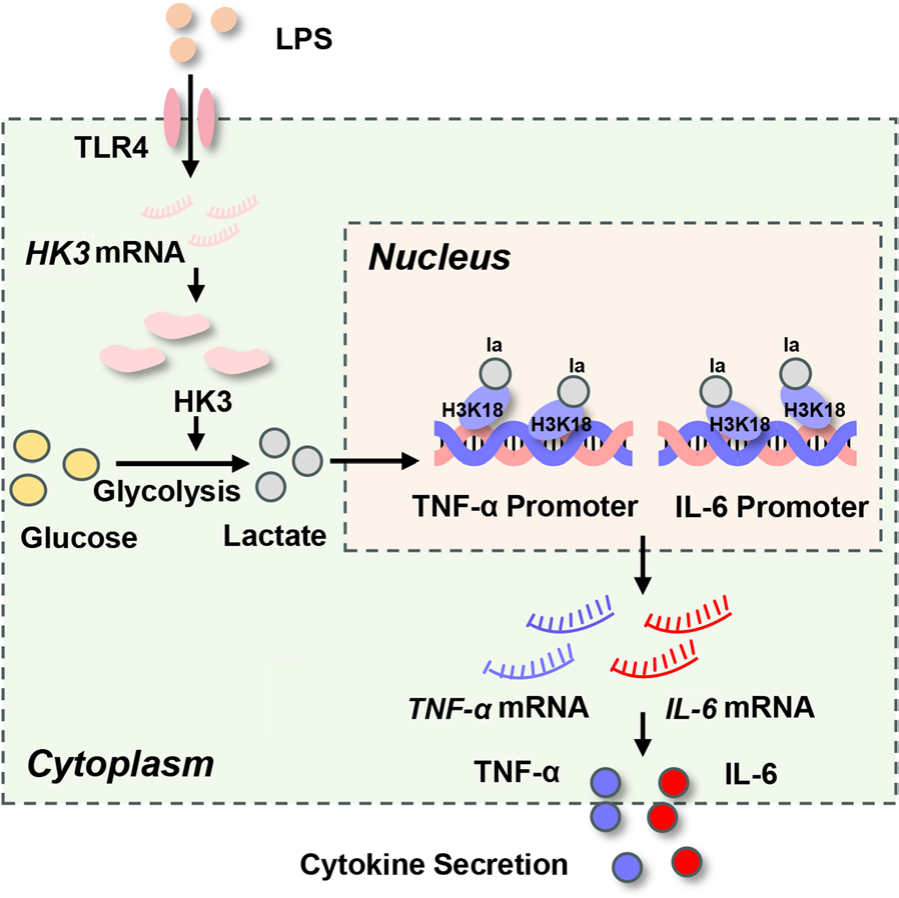
Schematic depiction of mechanisms underlying HK3 and sepsis. HK3 promoted cytokine production of monocytes by targeting metabolic reprogramming and histone lactylation in sepsis

In summary, our study demonstrates that HK3 plays a pivotal role in sepsis by promoting inflammatory cytokine production through its regulation of monocyte metabolic reprogramming and histone lactylation modifications (Fig. 8).

## Discussion

The HK family comprises four isoforms—HK1, HK2, HK3, and HK4—with distinct expression patterns and functions [14]. HK1 is ubiquitously expressed in brain, muscle, erythrocytes, etc. and binds to mitochondrial outer membranes to promote glycolysis [15]. HK2 is primarily expressed in insulin-sensitive tissues (adipose, skeletal muscle, heart, etc.) and tumor cells, being induced by growth factors and hypoxia [16]. HK3 is expressed in monocytes/macrophages and lungs, potentially involved in immunometabolism [17]. HK4 is liver- and pancreatic β-cell-specific, functioning as a glucose sensor to regulate insulin secretion and hepatic glucose metabolism [18]. All HK isoforms catalyze glucose phosphorylation to glucose-6-phosphate (G-6-P), serving as rate-limiting enzymes in glucose metabolism and glycolysis. Their differential tissue distribution, kinetic properties, and regulatory mechanisms enable precise control of cellular energy metabolism and biosynthesis [14] Through integrated GEO database mining and glycolysis-related gene set analysis, we identified that among HK family members, only HK3 and HK2 showed significant differential expression in septic patients' peripheral blood, with HK3 expression being higher than HK2. ROC curve analysis revealed HK3 as a novel metabolic checkpoint in sepsis patients' peripheral blood with excellent diagnostic value. During sepsis progression, metabolic reprogramming plays a central role in disease development and immune regulation. Peripheral blood metabolic checkpoints not only accurately reflect pathophysiological status but also provide new research directions for diagnostic biomarker development, prognostic evaluation systems, and targeted therapies. Traditional metabolic markers like lactate serve as key indicators of tissue hypoxia and mitochondrial dysfunction in sepsis patients, while their dynamic changes (e.g., ≥ 10% lactate clearance within 6 h) effectively predict treatment response and clinical outcomes [19–21]. As a key rate-limiting enzyme in glycolysis, HK3 is specifically highly expressed in monocytes and directly associated with sepsis-related immunometabolic reprogramming, thus being more capable of specifically reflecting the pathological process of this disease. In contrast, elevated lactate levels can be caused by a variety of non-sepsis factors, such as strenuous exercise, tissue hypoxia, liver insufficiency, or shock, resulting in its relatively low diagnostic specificity for sepsis [22, 23]. It was reported that pyruvate kinase M2 (PKM2), a glycolytic enzyme involved in metabolic reprogramming and gene transcription in immune cells, shows elevated serum levels correlated with disease severity and outcomes in sepsis patients [24]. In our study, the AUC of PKM2 was 0.785, which was lower than that of HK3 (AUC: 0.995), indicating that HK3 has better diagnostic value than PKM2. Our study innovatively identifies HK3 as a novel metabolic checkpoint with high diagnostic value in septic patients' peripheral blood, providing important theoretical basis for precise sepsis diagnosis and pathogenesis exploration.

Immune cells and microenvironment play crucial roles in sepsis progression [25, 26]. Using CIBERSORT algorithm, we analyzed immune cell infiltration in septic patients' whole blood, revealing significantly increased immune cell abundance, particularly monocyte infiltration, compared to controls. Single-cell RNA sequencing analysis revealed a significant increase in *HK3* expression in monocytes from the sepsis group compared to the control group.

In vitro experiments demonstrated that elevated HK3 correlates with monocyte hyperinflammation, while HK3 siRNA knockdown significantly suppresses this excessive inflammatory response. Circulating monocytes play complex and critical roles in sepsis pathogenesis. In the first place, through pattern recognition receptors (TLRs, NLRs) they identify PAMPs/DAMPs to initiate inflammatory responses [27]. In the next place, upon differentiation into dendritic cells or macrophages, they promote endothelial damage via antigen presentation, NO production, and protease release, contributing to microthrombosis, capillary leakage, and multiple organ dysfunction syndrome [25, 28]. Our work demonstrates significantly increased monocyte infiltration in septic patients' peripheral blood and elevated *HK3* expression in monocyte sepsis models, revealing *HK3*'s role in sepsis pathogenesis through regulating monocyte inflammatory responses. Our in vitro experiments found that low-dose LPS could induce the release of strong inflammatory factors but failed to upregulate *HK3* mRNA. This observation led us to hypothesize that *HK3* expression may be associated with more specific pathological states, and monocytes exhibit changes in *HK3* expression under high-intensity LPS stimulation. This finding further clarifies the potential functional window of HK3 in sepsis. It may be induced under certain disease states, participating in immunometabolic regulation and serving as an indicator of disease severity.

In sepsis, immune cells like monocytes exhibit significant metabolic alterations, particularly enhanced aerobic glycolysis [29]. This metabolic reprogramming closely correlates with inflammatory responses and cellular dysfunction, though its mechanisms remain unclear. Previous studies reported diverse regulatory functions of HK family in sepsis. It was demonstrated that HK2 plays an important regulatory role in glycolysis and immune function in macrophages [30]. Zhu et al. showed increased HK3 in alveolar epithelial sepsis models correlates with enhanced inflammation, oxidative stress and apoptosis [17]. However, how HK3 regulates monocyte inflammation in sepsis remains unexplored. Our study demonstrates that HK3-mediated metabolic reprogramming in monocytes leads to lactate accumulation. Beyond being a glycolytic byproduct, lactate serves as a signaling molecule for epigenetic regulation through histone lactylation—a novel protein post-translational modification linking metabolism with gene expression [31]. While previous studies reported lactate-modified HMGB1 enhancing endothelial permeability in polymicrobial sepsis [32] and H3K18 lactylation promoting anti-inflammatory Arg1 expression [33], we found HK3 promotes pro-inflammatory IL6 and TNF-α expression via regulating lactate accumulation and histone H3K18 lactylation to upregulate *IL-6* and *TNF-α* expression in monocytes. HK3 knockdown significantly suppresses metabolic reprogramming, lactate levels, and subsequent H3K18 lactylation/inflammatory gene activation. These findings elucidate HK3's crucial mechanism in monocyte inflammatory responses during sepsis pathogenesis. Our GEO data analysis found that both HK3 and HK2 are significantly elevated in the peripheral blood of sepsis patients, and whether the two play a synergistic role in the pathogenesis of sepsis requires further investigation.

Interestingly, we discovered that HK3 differentially regulates the production of the proinflammatory cytokines IL-6 and TNF-α. HK3 knockdown more strongly inhibits *IL-6* mRNA, and cytokine secretion compared to *TNF-α*, given both are regulated by HK3. Previous studies have reported that there is a differential regulatory mechanism for cytokine release [34, 35]. *IL6* gene locus might be more dependent on HK3-maintained levels of H3K18 lactylation for its full activation compared to the *TNF-α* locus. Consequently, HK3 knockdown, by reducing the H3K18 lactylation modifications, would have a more dramatic impact on *IL6* transcription than on *TNF-α*, which may be regulated by a more diverse set of epigenetic and transcription factors.

Our study has several limitations. Firstly, we only reported HK3's regulation on H3K18 lactylation—its effects on other histone lactylation sites (H3K9la, H3K14la, H3K23la, etc.) remain unclear. Furthermore, HK3's impact on non-histone protein lactylation warrants investigation to comprehensively understand its regulatory role. Another limitation is that we only validated the results in vitro. Further in vivo animal experiments can enhance the translational relevance of our conclusions.

In conclusion, our study demonstrates HK3 as a key regulator of monocyte glycolysis that mediates metabolic reprogramming to regulate inflammatory responses in sepsis pathogenesis, expanding understanding of HK3's role in sepsis. These findings provide novel insights into sepsis molecular mechanisms and important theoretical foundations for developing metabolism-targeted precision therapies against sepsis.

## Supplementary Information

Supplementary file 1.

Supplementary file 2.

## Data Availability

All data generated in this study are included in this article and supplementary file. The GEO data processing was performed using the publicly available online platform (https://www.bioinformatics.com.cn) as detailed in the Methods section. No custom code was developed or implemented for this study.
