# Supplementary material for: Hexokinase 3 promoted cytokine production of monocytes by targeting metabolic reprogramming and histone lactylation in sepsis
Source: Clin Epigenetics. 2026 Apr 10;18:103. doi: 10.1186/s13148-026-02129-6 (PMC13214176; doi:10.1186/s13148-026-02129-6)
Supplement: Supplementary file 2 [file 13148_2026_2129_MOESM2_ESM.docx]

**Figure 5D**


**Figure 6C**

**Figure 6E**





**Figure 7D**

**H3K18la**

**si-NC LPS+si-NC LPS+si-HK3**

**Histone H3**

**si-NC LPS+si-NC LPS+si-HK3**

**Figure 7F**

**LPS**

**IgG H3K18la Input**

**- + - + - +**

**250 bp**

**100 bp**

**IL-6**

**Marker**

**IgG H3K18la Input**

**- + - + - +**

**TNF-α**

**Lps**

**250 bp**

**100 bp**

**Marker**
